# Integrated Bioinformatics Analysis Exhibits Pivotal Exercise-Induced Genes and Corresponding Pathways in Malignant Melanoma

**DOI:** 10.3389/fgene.2020.637320

**Published:** 2021-02-18

**Authors:** Jun Zhu, Suyu Hao, Xinyue Zhang, Jingyue Qiu, Qin Xuan, Liping Ye

**Affiliations:** ^1^Administrative Office, Shanghai Basilica Clinic, Shanghai, China; ^2^Shuangwu Information Technical Company Ltd., Shanghai, China; ^3^School of Education, Hangzhou Normal University, Hangzhou, China; ^4^School of Physical Science and Engineering, East China University of Science and Technology, Shanghai, China; ^5^School of Sports Science and Engineering, East China University of Science and Technology, Shanghai, China; ^6^Department of Clinical Nursing, Minhang Hospital, Fudan University, Shanghai, China

**Keywords:** malignant melanoma, exercise, integrated bioinformatics analysis, Disease-free survival, prognosis

## Abstract

Malignant melanoma represents a sort of neoplasm deriving from melanocytes or cells developing from melanocytes. The balance of energy and energy-associated body composition and body mass index could be altered by exercise, thereby directly affecting the microenvironment of neoplasm. However, few studies have examined the mechanism of genes induced by exercise and the pathways involved in melanoma. This study used three separate datasets to perform comprehensive bioinformatics analysis and then screened the probable genes and pathways in the process of exercise-promoted melanoma. In total, 1,627 differentially expressed genes (DEGs) induced by exercise were recognized. All selected genes were largely enriched in NF-kappa B, Chemokine signaling pathways, and the immune response after gene set enrichment analysis. The protein-protein interaction network was applied to excavate DEGs and identified the most relevant and pivotal genes. The top 6 hub genes (Itgb2, Wdfy4, Itgam, Cybb, Mmp2, and Parp14) were identified, and importantly, 5 hub genes (Itgb2, Wdfy4, Itgam, Cybb, and Parp14) were related to weak disease-free survival and overall survival (OS). In conclusion, our findings demonstrate the prognostic value of exercise-induced genes and uncovered the pathways of these genes in melanoma, implying that these genes might act as prognostic biomarkers for melanoma.

## Introduction

Melanoma is an aggressive melanocytes-caused carcinoma (Precazzini et al., 2020). It can either locally invade surrounding tissues or metastasize throughout the overall body (Moser and Grossman, 2018). Because of the high mortality of melanoma, it is a major serious skin carcinoma, and also one of the fastest-growing malignant neoplasms in terms of incidence (Leiter et al., 2014). Proliferative melanoma (Magro et al., 2006), polypoid melanoma (Manci et al., 1981), primary skin melanoma (Maize, 1983), verrucous malignant melanoma (Steiner et al., 1988), pigment epithelioid melanoma (de Oliveira et al., 2020), mucosal melanoma (Postow et al., 2012), follicular melanoma (Hantschke et al., 2004), and non-melanoma cell differentiated melanoma (Madan and Lear, 2010) are the most problematic types of melanomas. Local resection is a conventional and basic clinical treatment strategy, but the prognosis is extremely poor (Foulds et al., 1987; Damato, 1993). Melanoma is not sensitive to chemotherapy since the efficacy of chemotherapy is relatively lower (Lee et al., 1995). Combined treatment performs better and exerts some certain significance on overcoming melanoma (O’Day et al., 2002). Gene therapy has been used in clinical practice (Heller and Heller, 2010), but it is non-directional, and the efficiency of gene transfer is refractory (Nakamura et al., 2002). Melanoma vaccine is a control method rather than a treatment method, and has demonstrated some limitations in the treatment of melanoma (Bystryn et al., 1992; Zhou et al., 1999). As for the prognosis, in the histopathological criteria, the thickness of tumor, mitosis rate and ulcers are considered to be the most important prognostic indicators (Azzola et al., 2003). At present, the incidence of melanoma in China is very low because of insufficient awareness of its severity (Wu Y. et al., 2020). It is therefore greatly important to accurately assess the progress of melanoma, and it is urgent to develop phase identification of molecular biomarkers for high-risk melanoma.

Regular exercise can decrease the risk of carcinoma and disease recurrence (Courneya, 2003). Studies have demonstrated findings on the potential role of exercise in the reduction of hypoxia and tumor immunity (Zhang et al., 2019). Some research indicates that exercise could ameliorate the physical function of carcinoma patients in terms of fatigue and life quality (Conn et al., 2006). Energy balance displays are essential in the prognosis of metastatic melanoma (Harvie et al., 2005). Additionally, exercise can directly affect the microenvironment of a tumor (Zhang et al., 2019). Numerous studies have shown that exercise could alter the tumor microenvironment and carcinoma-associated events, thus improving the structure and function of blood vessels (Zielinski et al., 2004). A poorly functioning vascular system can give rise to hypoxia of neoplasm, which conversely strengthens the invasiveness and stimulates metastasis of a tumor (Subarsky and Hill, 2003; Finger and Giaccia, 2010). Hypoxia is one of the key inducers in facilitating the transformation of melanocytes into tumor cells. When the transformation occurs, the rapid growth of melanocytes in the developing melanoma will heighten the oxygen demand and promote the generation of hypoxia, thus resulting in the angiogenesis driven by hypoxia-inducible factor-1α and the progress of dysfunctional tumor vasculature (Bedogni and Powell, 2009).

Recently, multi-center genomics, such as transcriptomics, proteomics, and high-throughput sequencing, have been widely used in various fields of life sciences. Many carcinoma-associated genes have been identified through comprehensive bioinformatics analysis, including breast cancer (Khan et al., 2020), lung adenocarcinoma (Selvaraj et al., 2018), and Diamond-Blackfan anemia (Khan et al., 2018), laying the foundations for the study of human tumors. In colorectal carcinoma, 31 hub node genes and their pathways have been identified through comprehensive bioinformatics analysis (Xu et al., 2004; Liang et al., 2016). These candidate genes and pathways can become therapeutic targets for colorectal cancer. In papillary thyroid carcinoma, new clinically relevant genes of papillary thyroid carcinoma are identified by analyzing four original microarray data sets (Liang and Sun, 2018). The comprehensive analysis of high-throughput omics data and clinical databases provides us with a good opportunity to discover new targets for cancer.

In this study, we introduced an integrated bioinformatics analysis as an effective method to evaluate the exercise-induced key genes associated with malignant melanoma. In the biological process (BP) group, the response of cells to immunity was revealed by Gene Ontology (GO) analysis. Pathway enrichment analysis data demonstrated NF-kappa B and Chemokine signaling pathways were two main enrichments. The top 6 hub genes Itgb2, Wdfy4, Itgam, Cybb, Mmp2, and Parp14 were screened and identified after protein-protein interaction (PPI) network analysis. These findings develop an understanding of the role of genes affected by exercise and provide a new method for analyzing the mechanism of other variable host factors on carcinomas.

## Materials and Methods

### Databases

The GSE62628 database^1^ was used to acquire microarray data including 10 datasets. The GSE62628 database was released on July 21, 2016. The database contains the gene expression profiles of two groups of exercise and non-exercise mouse melanoma tumor tissues. We identified five exercise group gene expression profiles, including GSM1530453 Exercise 300, GSM1530454 Exercise 301, GSM1530455 Exercise 306, GSM1530456 Exercise 309, and GSM1530457 Exercise 310. The five non-exercise gene expression profiles include GSM1530458 non-Exercise 326, GSM1530459 non-Exercise 327, GSM1530460 non-Exercise 329, GSM1530461 non-Exercise 330, and GSM1530462 non-Exercise 331. Malignant melanoma before or after voluntary exercise was included in the integrated analysis. The clinical meaning and function of the exercise-induced genes associated with melanoma were explored by The Cancer Genome Atlas (TCGA, https://www.cancer.gov/about-nci/organization/ccg/research/structural-genomics/tcga) database. The association between hub exercise-related gene expression and survival time in melanoma were analyzed by Gene Expression Profiling Interactive Analysis (GEPIA, http://gepia.cancer-pku.cn/) database. The hub exercise-related gene expression in melanoma samples and normal samples was also analyzed accordingly.

### To Identify the DEGs

Limma R package (Nagy et al., 2018) was applied to analyze the differentially expressed genes (DEGs). The cutoff standard was defined as | logFC| > 1 and adjusted *P* value < 0.05. Finally, the GEO2R website^2^ (Wu H. et al., 2020) was applied to analyze the DEGs.

### GO and Kyoto Encyclopedia of Genes and Genomes Pathway Enrichment Analysis

The molecular function (MF), BP, and cellular component (CC) were used in the GO analysis. Kyoto Encyclopedia of Genes and Genomes (KEGG) was applied to understand the function of genes or proteins. The GO and KEGG pathway enrichment analysis of DEGs was completed by the DAVID database (Dennis et al., 2003). The cut-off standard for both GO analysis and KEGG pathway enrichment analysis was adjusted *P*-value < 0.05.

### Construction of PPI Network and Analysis of Modular

A PPI network was constructed to validate the DEGs and also compare the interactions between or among these DEGs. We used the STRING database (Szklarczyk et al., 2017) and Cytoscape software (version 3.7.2; Wu X. et al., 2020) to visualize the PPI network. The obvious hub gene modules in the PPI network were screened by the Molecular Complex Detection (MCODE) plug-in in Cytoscape. The selection standards comprised: degree cutoff (number of connections with other nodes) ≥2; node score cutoff (the most influential parameter for cluster size) ≥2; and K-core (This parameter filtered out clusters that did not contain a maximally inter-connected sub-cluster of at least *k* degrees).

### Statistical Analysis

R software (version 3.4.2) was applied to analyze the derived data. The OS was evaluated by the Kaplan–Meier method. The log-rank test was applied to analyze the survival difference. *P* < 0.05 means significant statistic difference.

## Results

### GSE62628 Database Analysis

**FIGURE 1:**
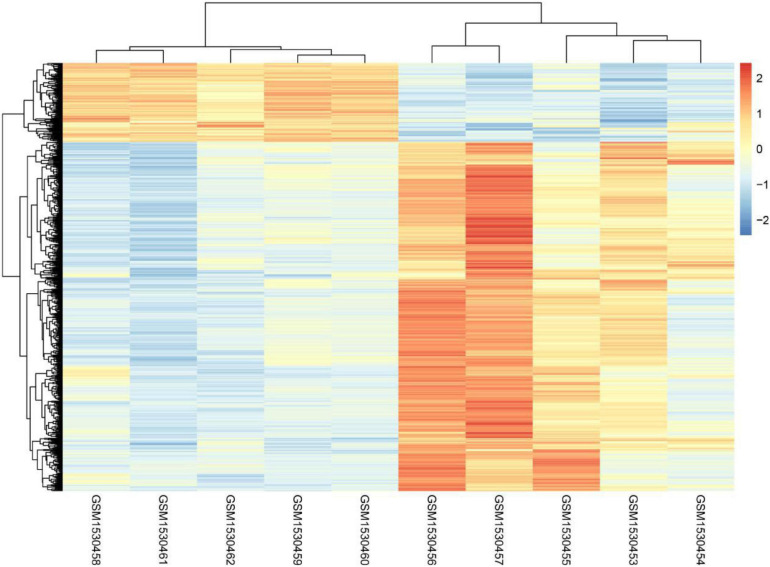
The heatmap analysis of GSE62628.

Even though it is well known that voluntary exercise could decrease both risk of carcinoma and the disease recurrence rate, the mechanisms regarding the protection provided by exercise are not yet clear. The present study analyzed the whole genome expression change of malignant melanoma before or after voluntary exercise. Finally, 1,285 genes with increased expression and 342 genes with decreased expression were screened and identified after voluntary exercise. Figure 1 displays the heatmap of the DEGs.

The present study analyzed the whole genome expression change of malignant melanoma before or after voluntary exercise, including 10 data sets (GSM1530454, GSM1530453, GSM1530455, GSM1530457, GSM1530456, GSM1530460, GSM1530459, GSM1530462, GSM1530461, and GSM1530458). We identified 1,285 up-regulated genes and 342 down-regulated genes after voluntary exercise. Red indicates that the DEG is up-regulated, blue indicates that the DEG is down-regulated, and white indicates that it is not expressed in one of the samples.

### GO Analysis of Exercise-Related Genes in Melanoma

**FIGURE 2:**
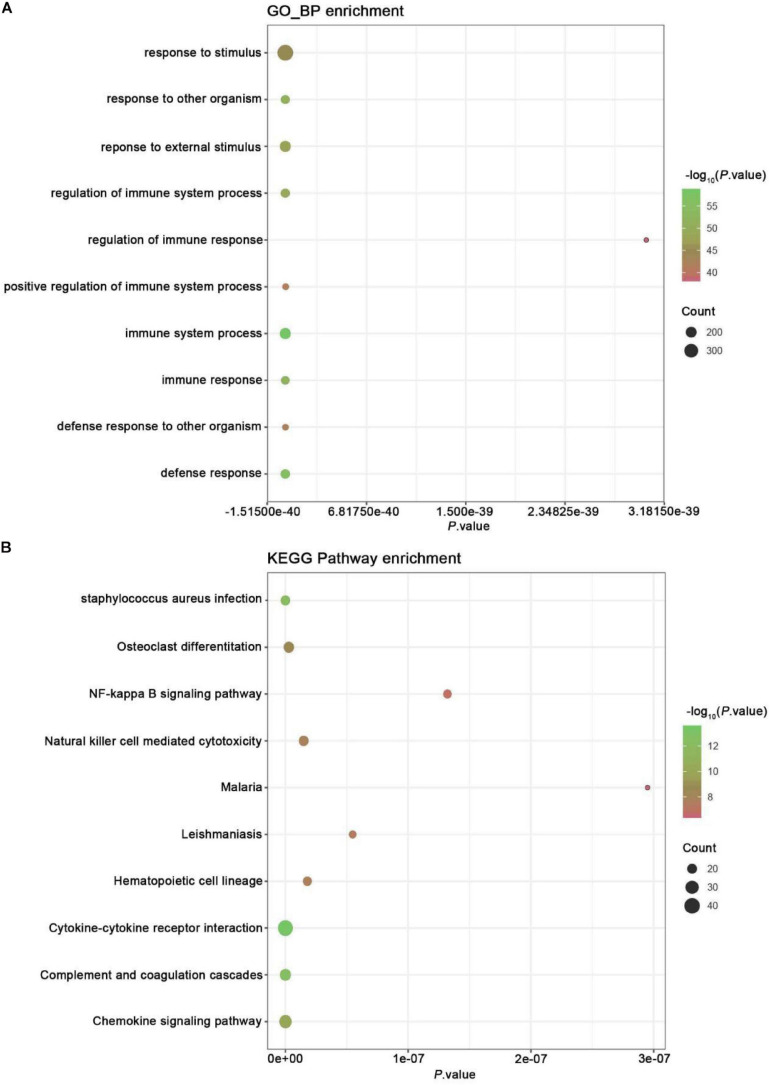
GO and KEGG pathway enrichment analysis. **(A)** GO analysis of all DEGs. The chart lists the richest Go terms of BP. **(B)** KEGG pathway analysis of all DEGs. The figure shows the most abundant KEGG pathway.

Gene ontology analysis was conducted to analyze the features of these DEGs (Figure 2A). Our analysis showed exercise related genes in melanoma were related to the modulation of self-defense response and defense to an exterior organism, immune response, immune system process, and response to a foreign stimulus.

### Signaling Pathway Enrichment Analysis

Kyoto Encyclopedia of Genes and Genomes pathway enrichment analysis was conducted to examine the mechanism of exercise-related genes in melanoma (Figure 2B). The data revealed that the identified DEGs were mainly enriched in pathways, like NF-kappa B, chemokine signaling, complement and coagulation cascades, cytokine-cytokine receptor interaction, hematopoietic cell lineage, leishmaniasis, malaria, cytotoxicity mediated by natural killer cell, osteoclast differentiation, and staphylococcus aureus infection.

The *X*-axis represents *P*-value; the *Y*-axis represents the GO-BP term or enriched pathway; color represents the *P*-value. The size of the dot represents the gene count.

### PPI Network Analysis of Pivotal Genes Amid DEGs

**FIGURE 3:**
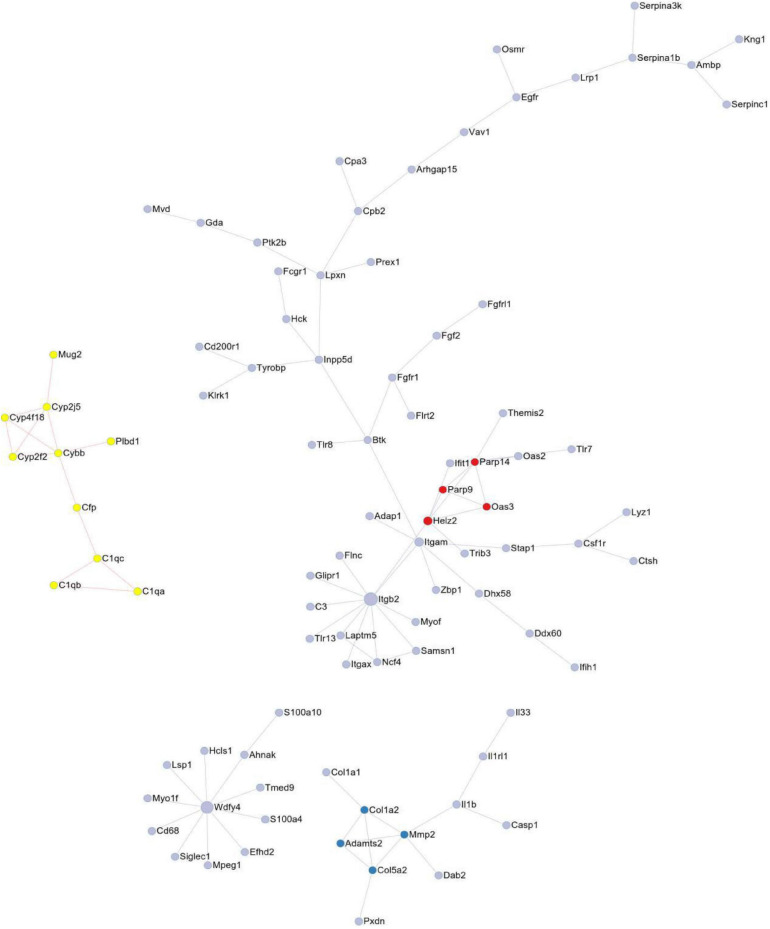
The protein-protein interaction (PPI) of DEGs exported from STRING is visualized using Cytoscape software.

We performed an analysis of the PPI network utilizing the STRING database to reveal the interaction among these exercise-related genes in melanoma (Figure 3). 92 DEGs comprising 92 nodes and 78 edges were screened after PPI network analysis. We verified 6 genes including Itgb2, Wdfy4, Itgam, Cybb, Mmp2, and Parp14 as hub genes with degrees > 5.

Protein-protein interaction network consisting of 92 nodes and 78 edges, and 6 genes were identified as hub genes with degrees >5, including Itgb2, Wdfy4, Itgam, Cybb, Mmp2, and Parp14. The node represents the gene; the edge represents the interaction between the two proteins.

### Exercise-Related Hub Genes Were Probably Dysregulated in Melanoma

**FIGURE 4:**
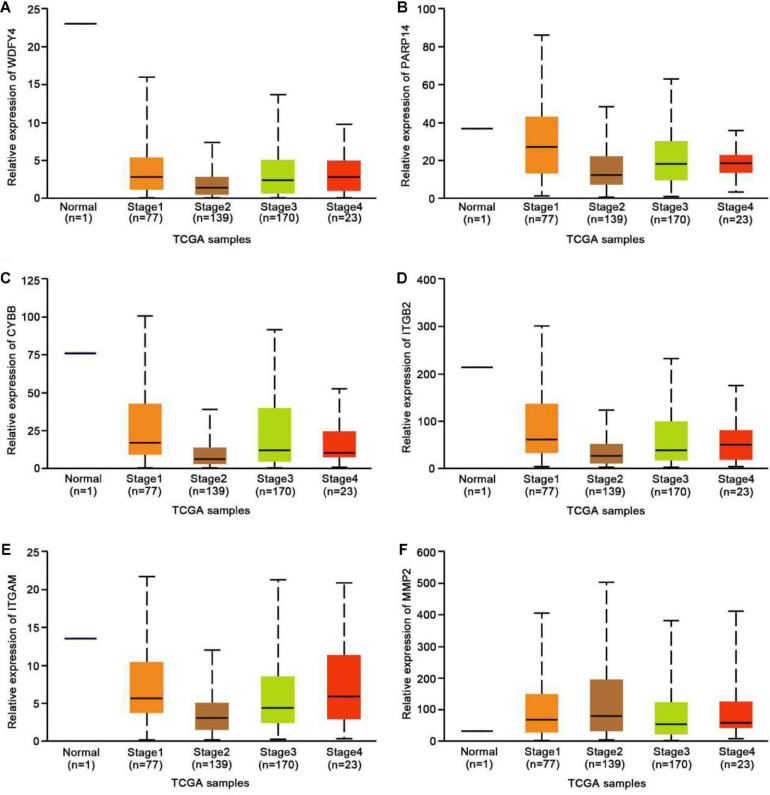
Expression of hub genes in different melanoma stages in the TCGA database. **(A)** The TCGA database was used to analyze the expression of WDFY4 in melanoma and normal samples. **(B)** The TCGA database was used to analyze the expression of PARP14 in melanoma and normal samples. **(C)** The TCGA database was used to analyze the expression of CYBB in melanoma and normal samples. **(D)** The TCGA database was used to analyze the expression of ITGB2 in melanoma and normal samples. **(E)** The TCGA database was used to analyze the expression of ITGAM in melanoma and normal samples. **(F)** The TCGA database was used to analyze the expression of MMP2 in melanoma and normal samples.

**FIGURE 5:**
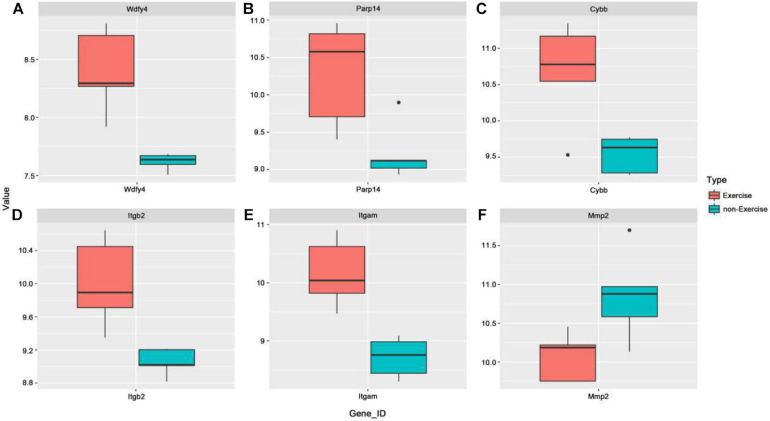
Expression of hub genes was analyzed in the GSE62628 database. **(A)** The GSE62628 database was used to analyze the expression of WDFY4 in exercise and non-exercise samples. **(B)** The GSE62628 database was used to analyze the expression of PARP14 in exercise and non-exercise samples. **(C)** The GSE62628 database was used to analyze the expression of CYBB in exercise and non-exercise samples. **(D)** The GSE62628 database was used to analyze the expression of ITGB2 in exercise and non-exercise samples. **(E)** The GSE62628 database was used to analyze the expression of ITGAM in exercise and non-exercise samples. **(F)** The GSE62628 database was used to analyze the expression of MMP2 in exercise and non-exercise samples.

To further explore the possible functional roles of exercise in melanoma, we also analyzed expression in melanoma and normal samples using the TCGA database. Figure 4A showed that decreased expression of Wdfy4 was demonstrated in stages 1–4 of melanoma samples but not normal samples. However, Wdfy4 was not differently expressed amid different stages of melanoma. Parp14 was reduced from stage 1 to stage 4 of melanoma samples compared to normal samples, and negatively correlated to the advanced stages of melanoma (Figure 4B). CYBB was decreased in stages 1–4 of melanoma samples compared to normal samples and ablated in stage 2 and stage 4 melanoma samples compared to that in stage 1 of melanoma samples (Figure 4C). Itgb2 was reduced in stage 1–4 of melanoma samples compared to that in normal samples and down-regulated in stage 2 and stage 3 of melanoma samples compared to that in stage 1 of melanoma samples (Figure 4D). Itgam was down-regulated in stage 1–4 of melanoma samples compared to that in normal samples and down-regulated in stage 2 of melanoma samples compared to that in stage 1 of melanoma samples (Figure 4E). However, our data revealed that MMP2 was up-regulated in stages 1–4 of melanoma samples compared to that in normal samples (Figure 4F). Nevertheless, MMP2 was not differentially expressed among different stages of melanoma. In addition, based on the GSE62628 database, we also analyzed the expression levels of the 6 hub genes before and after voluntary exercise. As shown in Figure 5, compared with non-exercise samples, the expression of Wdfy4, Parp1, CYBB, Itgb2, and Itgam in exercise samples increased. However, our data revealed that compared with non-exercise samples, MMP2 was down-regulated in exercise samples (Figure 5F).

### Exercise-Related Hub Genes Were Correlated to the Survival Time in Melanoma

**FIGURE 6:**
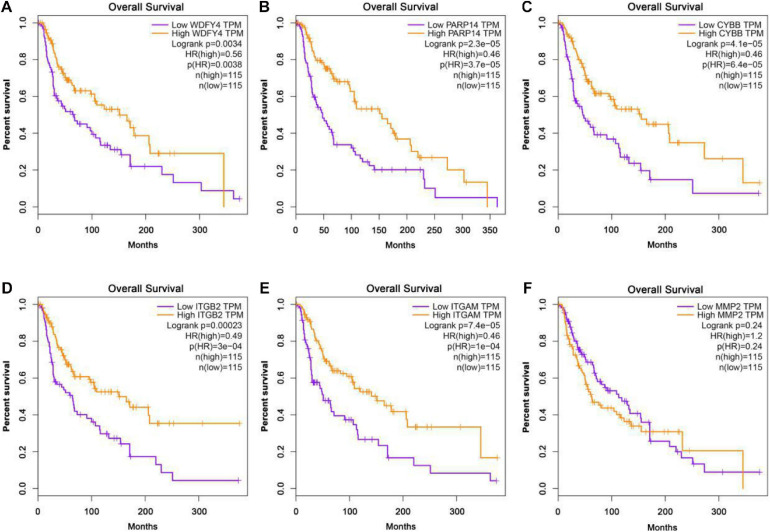
Overall survival (OS) analysis of hub genes in melanoma patients in GEPIA database. **(A)** OS of melanoma patients with the WDFY4 high expression level group (purple) and low expression level group (orange). **(B)** OS of melanoma patients with the PARP14 high expression level group (purple) and low expression level group (orange). **(C)** OS of melanoma patients with the CYBB high expression level group (purple) and low expression level group (orange). **(D)** OS of melanoma patients with the ITGB2 high expression level group (purple) and low expression level group (orange). **(E)** OS of melanoma patients with the ITGAM high expression level group (purple) and low expression level group (orange). **(F)** OS of melanoma patients with the MMP2 high expression level group (purple) and low expression level group (orange).

**FIGURE 7:**
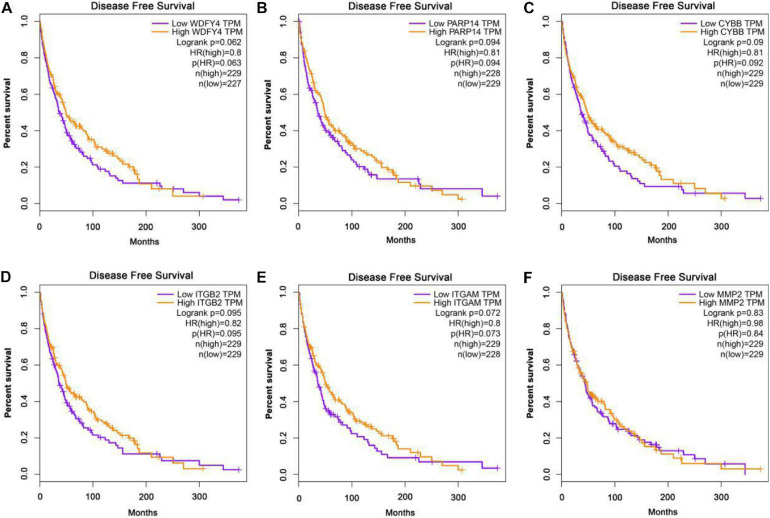
Disease-free survival (DFS) analysis of hub genes in melanoma patients in GEPIA database. **(A)** DFS of melanoma patients with the WDFY4 high expression level group (purple) and low expression level group (orange). **(B)** DFS of melanoma patients with the PARP14 high expression level group (purple) and low expression level group (orange). **(C)** DFS of melanoma patients with the CYBB high expression level group (purple) and low expression level group (orange). **(D)** DFS of melanoma patients with the ITGB2 high expression level group (purple) and low expression level group (orange). **(E)** DFS of melanoma patients with the ITGAM high expression level group (purple) and low expression level group (orange). **(F)** DFS of melanoma patients with the MMP2 high expression level group (purple) and low expression level group (orange).

The GEPIA database was applied to analyze the links between exercise-related hub gene expression and survival time in melanoma. As shown in Figure 6, melanoma patients with high expressions of Wdfy4, Parp14, Cybb, Itgb2, and Itgam were found to be associated with the longer OS time in melanoma. However, the dysregulation of MMP2 was not correlated to melanoma. The DFS time analysis revealed that there was a similar trend that high expressions of Wdfy4, Parp14, Cybb, Itgb2, and Itgam were correlated to longer DFS time in melanoma (Figure 7).

## Discussion

Melanoma data from most countries indicate that the incidence of this condition is increasing rapidly (Leiter et al., 2014). Pathogenic events in melanoma can be triggered by molecular mechanisms (such as point mutations, deletions, and translocations) or epigenetic mechanisms (such as microRNA expression and promoter methylation), thus resulting in the activation of oncogenes or the inactivation of neoplasm suppressor genes (Hussein et al., 2003; Lee et al., 2014). Studies have shown that the methylation of the RASSF1A promoter is closely related to susceptibility to melanoma (Guo et al., 2019). The variant copies of the GGC repeat sequence in the NPAS2 clock gene likely led to the tumorigenesis of melanoma (Franzoni et al., 2017). It has also been found that microRNA can control gene expression after transcription, thereby regulating various cell signaling pathways in the tumorigenesis and development of melanoma (Philippidou et al., 2010). Several reports have revealed that the change of function for tumor non-infiltrating lymphocytes caused by exercise can slow down the progression of melanoma, indicating that the routine practice of moderate-intensity exercise may be an effective potential treatment strategy (Dos Santos et al., 2019). It is worth noting that research also found that physical activity and melanoma seemed to have a positive correlation, which might be caused by risk factors related to ultraviolet radiation (Young, 2009).

In the current study, a total of 1,627 DEGs were verified between the voluntary exercise-related melanoma samples and control samples, including 1,285 genes with increased expression and 342 genes with decreased expression. It was found that these DEGs were enriched for NF-kappa B and Chemokine signaling pathways and immune response. NF-κB is increasingly recognized as a vital participant in many steps of carcinoma initiation and development. Similarly, the role of NF-κB was also shown in colon carcinoma, stomach carcinoma, and liver carcinoma (Grivennikov and Karin, 2010). Studies have shown that NF-κB is one of the main factors that controlled the resistance of pre-tumor and malignant cells to apoptosis-based tumor monitoring and regulated tumor angiogenesis and invasion (Wang et al., 2009).

Previous studies have shown that multiple genes in melanoma cells, such as Wnt5a, MELK, and PTX3, can trigger the NFκB signaling pathway, thereby migrating and invading melanoma (Janostiak et al., 2017; Barbero et al., 2019; Rathore et al., 2019). Chemokines were considered to be an important multifunctional cytokine in modulating the proliferation, invasion, and migration of neoplasm in an autocrine or paracrine manner. Additionally, chemokines were reported to mediate the tumorigenesis and development of numerous carcinomas, comprising breast carcinoma, prostate carcinoma, lung carcinoma, colorectal carcinoma, and melanoma (Lazennec and Richmond, 2010). Endogenous Wnt5a in cells exert an immunomodulatory effect on melanoma by secreting chemokines (Barbero et al., 2019). Chemokines were shown to essentially display in the development of melanoma. The growth and progress of carcinoma were demonstrated to be related to immunosuppression. Cancer cells were liable to motivate specific immune checkpoint pathways with corresponding immunosuppressive functions. Melanoma is a complex malignant tumor with diverse genomes (Lin et al., 2008). New genes and signal pathways involved in the pathogenesis are constantly being discovered. Immune checkpoint inhibitors have greatly changed the treatment options for melanoma. In recent years, research on the mechanism of immune regulation in melanoma has led to the development of many successful and innovative pharmaceutical preparations (Azijli et al., 2014).

The PPI network analysis revealed that 6 hub genes, comprising Itgb2, Wdfy4, Itgam, Cybb, Mmp2, and Parp14, might be key candidate genes related to the pathogenesis of melanoma. Itgb2 has been identified as a key oncogene in many human cancers, such as clear cell renal cell carcinoma, high-grade serous ovarian cancer, and lung adenocarcinoma. It was found that high expression of Itgb2 in triple-negative breast carcinoma exerted effects on the prognosis of patients. Wdfy4 regulates B cells through atypical autophagy and is genetically related to the susceptibility of systemic lupus erythematosus (SLE) of various races (Yuan et al., 2018). It has been clinically found to be significantly associated with Wdfy4 and patients with myopathy dermatomyositis (Kochi et al., 2018). Studies have shown that TGAM is not a general autoimmune gene, it is a risk factor for SLE, and is genetically related to a variety of autoimmune diseases. CYBB, and the NADPH oxidase gene, show gender-specific differential expression in multiple sclerosis (Cardamone et al., 2018). About 70% of patients with chronic granulomatous disease (CGD) have a mutation in the CYBB gene on the X chromosome (Eguchi et al., 2018). MMP2 is involved in the development of extracapillary proliferative diseases (Phillips et al., 2017). PARP14 can promote pancreatic cancer (PC) cell proliferation, anti-apoptosis, and GEM resistance, highlighting its potential role as a therapeutic target for PC (Yao et al., 2019). PARP14 was shown to be a newly produced drug target for carcinomas (such as diffuse large B-cell lymphoma, multiple myeloma, prostate carcinoma, and hepatocellular carcinoma) and allergic inflammation (Qin et al., 2019). These studies show that these hub genes are highly related to the progression of carcinoma and autoimmunity. In the present study, we found that Wdfy4, Parp14, Cyb, Itgb2, and Itgam were related to the prognosis of patients. Our data also revealed that the expression of the hub genes and the tumor stages of melanoma patients correlated, indicating these hub genes were promising biomarkers and targets for the diagnosis of melanoma patients and treatment. However, further experiments toward the parts of these genes need to be validated.

Based on the TCGA database, we found that Wdfy4, CYBB, Itgb2, and Itgam were down-regulated and MMP2 was up-regulated in melanoma samples compared with normal samples. Based on the GSE62628 database, we found that compared with non-exercise samples, Wdfy4, Parp1, CYBB, Itgb2, and Itgam were up-regulated and MMP2 down-regulated in the exercise samples.

Previous studies have shown that exercise is related to cancer recurrence and a significant reduction in mortality, and exercise intervention is beneficial to cancer patients. Our results showed that exercise inhibited high-expressed genes in melanoma, while it promoted low-expressed genes. Exercise might inhibit the progression of melanoma by inhibiting the expression of proto-oncogene and promoting the expression of tumor suppressor genes. In future research, we will analyze the expression level of the hub gene and its prognostic value in clinical samples. We will conduct more experiments in the future that explore knockdown and overexpression in the 5 hub genes of cell lines and mouse models, to evaluate their characteristics during the development of melanoma. In addition, the regulation mechanism of exercise on melanoma will also become the direction of our next research.

In short, comprehensive bioinformatics analysis has provided a research method by analyzing 3 databases to screen the exercise-induced key genes of melanoma and we found that several pathways have been changed. The 5 hub gene expression was related to the clinical outcome of melanoma patients. These findings indicate that the selected candidate genes along with their related pathways might serve as therapeutic targets for melanoma, and comprehensive bioinformatics analysis might be considered a new paradigm to guide the study of the interaction between lifestyle and disease. Moderate exercise is essential to improve the outcome of cancer patients, and exercise might be a promising treatment option for melanoma.

## Data Availability Statement

Publicly available datasets were analyzed in this study. This data can be found here: https://www.ncbi.nlm.nih.gov/geo/query/acc.cgi?acc=GSE62628.

## Author Contributions

JZ, SH, and XZ: conception and design. JQ, LY, and QX: development of methodology. JZ, SH, and JQ: analysis and interpretation of data. XZ, JQ, and QX: writing, review, and revision of the manuscript. All authors contributed to the article and approved the submitted version.

## Conflict of Interest

SH was employed by company Shuangwu Information Technical Company Ltd, China. The remaining authors declare that the research was conducted in the absence of any commercial or financial relationships that could be construed as a potential conflict of interest.

## Footnotes

1 https://www.ncbi.nlm.nih.gov/geo/query/acc.cgi

2 https://www.ncbi.nlm.nih.gov/geo/geo2r/?acc=GSE62628

## Abbreviations

GO: Gene ontology
PPI: protein-protein interaction
TCGA: The Cancer Genome Atlas
GEPIA: Gene Expression Profiling Interactive Analysis
DEGs: differentially expressed genes
KEGG: Kyoto Encyclopedia of Genes and Genomes
MF: molecular function
BP: biological process
CC: cellular component
MCODE: Molecular Complex Detection
BiNGO: Plug-in Biological Networks Gene Oncology tool
SLE: systemic lupus erythematosus
CGD: chronic granulomatous disease
PC: pancreatic cancer.
